# An Inexpensive Unmanned Aerial Vehicle-Based Tool for Mobile Network Output Analysis and Visualization

**DOI:** 10.3390/s23031285

**Published:** 2023-01-23

**Authors:** Vittorio Buggiani, Julio César Úbeda Ortega, Guillermo Silva, Jesús Rodríguez-Molina, Diego Vilca

**Affiliations:** 1 Department of Telematics and Electronics Engineering, Universidad Politécnica de Madrid, 28040 Madrid, Spain; 2 Secondary RADAR and Identification, Friend or Foe Section, Indra Sistemas, 28108 Alcobendas, Spain

**Keywords:** Unmanned Aerial Vehicle, mobile network, Cyber-Physical System

## Abstract

Usage of Unmanned Aerial Vehicles (UAVs) for different tasks is widespread, as UAVs are affordable, easy to manoeuvre and versatile enough to execute missions in a reliable manner. However, there are still fields where UAVs play a minimal role regardless of their possibilities. One of these application domains is mobile network testing and measurement. Currently, the procedures used to measure the main parameters of mobile networks in an area (such as power output or its distribution in a three-dimensional space) rely on a team of specialized people performing measurements with an array of tools. This procedure is significantly expensive, time consuming and the resulting outputs leave a higher degree of precision to be desired. An open-source UAV-based Cyber-Physical System is put forward that, by means of the Galileo satellite network, a Mobile Data Acquisition System and a Graphical User Interface, can quickly retrieve reliable data from mobile network signals in a three-dimensional space with high accuracy for its visualization and analysis. The UAV tested flew at 40.43 latitude and −3.65 longitude degrees as coordinates, with an altitude over sea level of around 600–800 m through more than 40 mobile network cells and signal power displayed between −75 and −113 decibels.

## 1. Introduction

In recent years, Unmanned Aerial Vehicles (UAVs, also referred to as drones or Remotely Piloted Aircrafts, depending on their work procedures) have improved their capabilities and performance, while at the same time becoming more economical and easier to use. It has been proven that UAVs can provide a high degree of usefulness in application domains like agriculture [1], nature monitoring [2], infrastructure maintenance [3] and, generally speaking, secure information transfer among remote locations and devices [4] and Internet of Things environments where data collection and inference of knowledge are of major importance [5]. Indeed, UAVs have a collection of features that make them useful for environments where obtaining information from a wide area in a fast and responsive manner is of major importance: (a) they have a high degree of mobility (and therefore, they can perform manoeuvres and travel to areas where other bigger devices or humans might not be able to go, due to distance, positioning or how hazardous the environment is [6]); (b) they tend to be inexpensive to acquire and use (and can be employed without requiring large amounts of economic resources); (c) they are portable (therefore, they can be taken from/to different places without high requirements in space or storage room); (d) they can provide large amounts of data in a comparatively small amount of time (so they come in handy for any application that involves data, even in a big-data-like fashion [7]); (e) they can be used both in a piloted and autonomous manner (so they can be left to perform tedious tasks without the constant need of supervision). Despite the diversification in the usage of UAVs and the advantages that come with them, there are some application domains where UAVs have yet to become as popular as other hardware devices. For example, Renewable Energy Sources (RESs) can benefit from their monitoring features to detect solar panels with defective or underperforming cells [8] or to track and control cattle in sparsely populated areas [9], but, unfortunately, many application domains, for now, do not UAVs to the extent where it becomes clear how significant the benefits they can provide are.

One of these areas with great impact on society is mobile networking. As it is widely known, the usage of smartphones and mobile phones has dramatically increased during the last two decades, and therefore, the need to have a mobile network that these terminals can rely on as a trustworthy and that have fully functional deployment is often taken for granted. This necessity, and how it is satisfied by mobile network operators, has become a major business in Information and Communication Technologies (ICT). However, such an important system implies that it must have very high standards in terms of performance. Usually, mobile networks are expected to offer access to the web and provide connectivity to smart phones in a fast and trustworthy way, so that it can offer a satisfactory user experience in terms of web access and communication. Guaranteeing such output, however, demands a great number of resources to be put into the system for several tests aimed at attaining knowledge regarding network performance parameters. Among the latter, the monitoring and reconnaissance of the status and capabilities of such networks in a particular space is one of critical importance, as it will define how usable the mobile network is and what services it can provide.

### 1.1. Mobile Networks, Unmanned Aerial Vehicles and Their Synergies

As far as mobile network technologies are concerned, the need for solutions oriented towards acquiring data that can be used by the owners of the facilities and equipment used to offer mobile communications is of major importance. Data are required to have a clear view of how powerful network signals (3G, 4G and others) are in a specific area, location or even large structure (for example, a commercial aircraft). Depending on signal power figures, it might be required to redesign the future deployment of the communication infrastructure. Typically, measurements are taken by a team of trained staff (technicians, engineers) tasked with obtaining ground data regarding signal strength in different coordinates in an area. This team is deployed in the location of interest and, by using their equipment, they can obtain the measurements that are being sought. While such a procedure ensures that some information will be retrieved, it is flawed due to several reasons: (a) the area that can be covered by a team of people is restricted in terms of size and time; (b) the costs of such a procedure tend to be rather large; (c) there are multiple zones that, due to the nature of the equipment used, cannot be covered or are done so in a minimal way. Overall, although making use of trained staff can provide a certain quantity of measures, in the end, it cannot offer the same level of data quantity and quality that a free flight throughout a three-dimensional volume could offer, as the team deployed for such a purpose is unlikely to reach every point of a 3D space, or if they manage to do so, it will require a vast amount of resources in terms of time and budget. Our proposal puts forward an improvement, which is explained in the very next section.

### 1.2. Contributions of the Paper

This paper puts forward a system where several hardware and software components work together in an interweaved manner despite their distributed and heterogeneous nature. Considering the design, development and testing works carried out, and the solutions that are available in the existing published literature, it is the authors’ opinion that the paper provides the following contributions:

1. The usage of a UAV as a tool to take measurements from wireless networks in mobile communications. The usage of such a solution offers several advantages over the existing procedures: (a) it is faster to deploy a UAV or even a swarm of them than having teams of technicians and engineers performing measurements; (b) it is a far cheaper solution than having the aforementioned teams perform a similar functionality; (c) the mobility, positioning and speed that a UAV equipped with the correct sensors would have greatly surpasses what humans could perform, even with their set of measurement equipment. It must also be considered how UAVs can be used in locations that would be impossible for humans to access, or in a hazardous environment that could put human lives at risk. This tool not only performs extremely accurate measurements of 4G mobile networks, but also provides a way to visualize them and effectively offers a front-end Graphical User Interface (GUI).
2. Usage of Galileo as a high accuracy Global Navigation Satellite System (GNSS) for the UAV that has been built. Galileo presents several features that, as will be explained in Section 3, make it a desirable option as a GNSS system to both provide accurate positioning of the UAV and exact readings from mobile networks. Therefore, hardware that is capable of establishing communications based on Galileo services has been put to use.
3. A UAV built from scratch for the purpose of high-accuracy mobile network signal measurements: the UAV that has been built for the purposes of this paper has been done so from scratch. This was necessary due to several reasons. While a commercial solution equipped with the required sensors and a Galileo-enabled microcontroller would also be viable, it was chosen to use the presented UAV because the authors had tighter control over what was added to the UAV by mounting up the components themselves. This became especially important with the controller (Navio2) used with the UAV, as the authors were able to set what kind of hardware could use Galileo as the GNSS of choice and make use of its features.

### 1.3. Paper Structure

This paper is organized as follows: an introduction with the main themes of the paper has already been provided. The following section contains a study of the published literature and developments where solutions close to the one that is presented here are described and/or put to use. In addition to the study, the most important major issues have been included in this section as well. Section 3 describes the kind of system that has been conceived, along with its main features and how it tackles the most important open issues that have been found in the literature, to an extent. Section 4 contains the implementation works that have been carried out with the aim of providing a prototype that can prove the ideas that have been put forward in the previous section of the paper. Measurements obtained and how they are processed are other pieces of information contained here. Section 5 contains the conclusions and future works to be performed to improve the solution. Bibliographical references close the paper.

## 2. Related Works

The existing literature reflects on the many usages that UAVs have for different application domains, as well as how they can be used with regards to wireless communications. However, to the best of our knowledge, there have been minimal to no attempts at using the Galileo GNSS combined with an open-source UAV that is intended for procedures resembling what is put forward in this paper.

### 2.1. Study of the State-of-the-Art

It was described in [10] how a UAV can be used to obtain measurements related to Antennas Under Tests (referred to as AUT). The authors developed a methodology that, considering major parameters (distance from the antenna, its aperture, the operating wavelength, etc.), made use of a UAV to take in situ far-field measurements. The work developed comprised of five different subsystems (Transmitting, Receiving, Positioning, Recording and Data-Processing subsystems) and a simulation framework used for three of the most frequent use cases (probe in an elevated range, probe mounted on a UAV and a misalignment error analysis). The reviewed paper proved that there was interest behind the idea of using UAVs for wireless network measurements, but it provided a simulated environment for some tailored use cases, instead of offering an actual prototype with mobile network data collected from a real environment. No comparative measurements or time usage information were provided, as opposed to our own paper.

Another similar example was shown in [11], where an “antijam and accurate antenna parameters’ measurement framework” named A3PNet was put forward. It was cited how the measurement of the antenna down-tilt angle of mobile communication base stations was regarded as a critical task for academia and industry, and how UAVs could be used for that purpose. Measurement enhancement in an unfavourable environment (i.e., wind at certain altitude destabilizes the UAV used for antenna-related measurements) was the main topic of the reviewed paper, as (a) an Adaptive Feature Recovery Module (AFRM), (b) a Contour Modulation Strategy (CMS) and (c) an Antenna Down-tilt Measurement Module (ADMM) were introduced for the sole purpose of improving an antenna attitude and performance. In this context, UAVs were expected to be used as autonomously as possible with next to no human intervention. The work presented was remarkable in one sense, but it had a different purpose than the one presented in our paper (an inexpensive open-source tool to perform reliable mobile network measurements, making use of Galileo as the GNSS).

It was described in [12] how it is desirable to have a visual measurement system that can be used for Autonomous Aerial Refuelling in UAVs. It was described how UAVs of relatively large sizes can fly in an autonomous manner when they are performing tasks, but, unlike manned aircrafts, they lack aerial refuelling capabilities that would enable them to perform longer missions. To deal with this challenge, a Virtual Reality-based simulation environment was built, where visual markers and a camera were set in the simulated receiver UAV and the tanker aircraft, respectively. By making use of marker detections and point matching capabilities, the authors of the reviewed paper made a pose estimation that enabled them to demonstrate their hypothesis. The V-Realm Builder platform in MATLAB was used to create the simulated environment. Tests were carried out in a hardware-in-loop system to understand the feasibility of the proposed system. Overall, this piece of research showed how UAVs could be used in application domains that were traditionally kept for other larger, more complex aircraft or devices, but the scope of this paper was significantly different from the solution put forward in our own research and development works.

A similar orientation could be found in [13]. It was shown by the authors of this paper how a UAV could be employed to measure different kinds of parameters (temperature, humidity, thermal noise from the UAV, etc.), to the point that a UAV could be used as a Mobile Measurement Platform (MMP). However, it must be considered how these measurements were not as accurate as expected, since they were obtained in an indirect manner. Nevertheless, an example of the possibilities that UAVs offer when used as MMPs, using a UAV as a support system for three-dimensional reconstruction of archaeological files, was described. This piece of research proved that, as shown in our own study, UAVs could be used for purposes that go beyond mere camera visualization and collect data from a collection of different installed sensors if required, but there is no explanation on how a system for mobile network signal measurement could be built, nor the ancillary subsystems required to visualize the collected data and infer information from. Furthermore, no information has been provided on the set of user requirements that would be needed to create this kind of system.

Other research works of interest include those shown in [14]. As far as the research in this paper is concerned, the authors of this paper made use of a UAV to perform several operations, such as (a) conducting Air-to-Ground (AG) measurements for UAV vertical flights, (b) the Path Loss Exponent (PLEs) and height impact factor, (c) a proposed novel Autocorrelation Function (ACF) model of shadowing and (d) an analysis of Average Fade Duration (AFD) and Level Crossing Rate (LCR) parameters. To perform the AG measurements, a system composed by a DJI N3 six-rotor UAV (which was equipped with several hardware components used for data collection, like a GPS antenna, a telemetry module, test and antenna transmitters and a battery to feed power to the UAV), a test transmitter, a signal analyser, one laptop, and antennas was built. This research proved that UAVs could be used in an effective manner to gather information regarding channel measurements, but its focus was oriented towards the usability of UAVs as mobile base stations, thus differing from providing a full-fledged interface that could be offered as a user-friendly solution that provides detailed information with regards to mobile network signals.

Other kinds of measurement activities can also be carried out by UAVs, as described in [15]. In this case, the system that was developed had two main parts: a UAV platform consisting of an oil-powered unmanned helicopter SU-H2M with a maximum payload of 45 kg and a cruising speed of 60 kilometres per hour, and an aeromagnetic measurement equipment that had five parts: (a) a high-precision potassium pump magnetometer; (b) a three-axes fluxgate manometer; (c) an inertial navigation module; (d) an altimeter; (e) a data collector module with a data processing platform. The results showed that this system could be used to detect anomalies in specific geological locations, and a map with aeromagnetic data could be created with a very high degree of accuracy. As before, this paper proved that UAVs could take measurements in a reliable manner for a plethora of different application domains. However, the scope of this research was focused on aeromagnetic measurements rather than the characteristics of mobile network signals, which is a completely different application domain. In addition, there was no development of a GUI that showed, in a user-friendly manner, the results obtained from the signal.

A similar purpose was the focus of the research represented in [16], but instead of monitoring features or parameters related to wireless communications or man-made deployment of technology, it was *Phaeocystis globosa* alga (defined in the paper as a “*unique causative species of harmful algal blooms, which can form gelatinous colonies*”) the element being monitored. Blooms of this alga were sought, and their biomass was calculated according to the data collected by the UAV. These parameters became known based on in situ remote sensing reflectance, which was measured over a large distance range. Once the UAV had collected the data, it was used for the purposes described in the reviewed paper. While the scope of this reviewed piece of research was different compared to the one put forward in our paper, it again shows how UAVs can be used for measuring a significant diversity of parameters depending on the sensors they have been equipped with, as well as providing extra access and mobility to regions that could not be accessed with human operators or regular devices.

The work shown by Burak Ede et al. [17] described what the authors defined as: large-scale statistical modelling of air–to–air wireless UAV channels. This statistical modelling was created by using, among other features, propagation characterization of wireless communications enabled by Software Defined Radio (SDR) capabilities. As far as testing was concerned, two commercially available hexacopters were used as the transmitter and the receiver; both were equipped with a mounted channel measurement box. The purpose of the study fell close to the application domain that we have defined for our proposal, but there are some significant differences: the solution put forward by us is more oriented towards mobile network signal measurements, rather than modelling of wireless channels, and an end-user GUI is provided for better understanding the collected information. Furthermore, communications in the reviewed paper followed an A2A pattern, whereas the information is displayed in a GUI running on a computer in our study, thus using an Air-to-Ground (A2G) paradigm.

UAVs also play a major role in electromagnetic coupling measurements, as it was shown in [18]. The authors of this paper described how UAVs operate in congested wireless environments that, when used in certain application domains (i.e., mobile base stations) exacerbate electromagnetic compatibility (EMC) and/or electromagnetic interference (EMI). They claim that: “*To avoid undertesting and overtesting in EMC measurements, there is a strong need for a computational tool that can guide experimental measurements by predicting and quantifying the frequency and the orientation that causes the maximum electromagnetic coupling to a complex DUT* [Device Under Test]”. To accomplish this, a simplified model was created where a UAV was represented by a 220 millimetres edge length with four wires attached to the patch’s corners, making a 45 degrees angle with the edge of the square patch. A Characteristic Mode Analysis (CMA) was performed afterwards, with modal significance, currents and field/radiation patterns for the simplified model being studied. Overall, this piece of research touched on a compelling topic that is of great interest when having several devices working together with wireless communications, but its scope and purposes were significantly different from the ones put forward in our study.

It was described in [19] how multi-temporal UAV-obtained data could be used for measuring even rill and inter-rill erosion on the European loess belt. The usage of Digital Surface Models (DSMs) obtained as data acquired from a UAV was put forward to evaluate these erosion levels and take any required action, which could be as accurate as having less than 1 centimetre error in the imagery collected. The tool used for data acquisition was the UAV Falcon 8 (which received this name from being an octocopter), which, due to its hardware features (active stabilizing camera, capability to record data, camera with a zoom lens, etc.) was deemed suitable for this task. Furthermore, a Terrestrial Laser Scanning (TLS) device was used for further accuracy and reliability in error assessments of the aerial data. By means of this tool, it was determined with high precision what kind of erosion takes place during rainfall days in spring or thunderstorms in summer, reaching the conclusion that the greatest erosion volumes occur because of rill erosion instead of inter-rill erosion. As with previous papers, while the objective of this one was set in a rather different environment than the one used in our own study, it proved that UAVs can be used for different purposes that go beyond monitoring with a portable camera. 

More related to concepts of smart farming and precision agriculture, the paper authored by Thomas Arnold et al. [20] discussed the integration of “*an airborne multi-spectral imaging sensor which is able to simultaneously capture three visible and three near-infrared channels*” in a UAV. To accomplish this goal, a multi-channel camera system (Condor-1000 MS5 5 CCD) was mounted as a peripheral in the CAMCOPTER S-100 UAV. It was explained how one of the main interests in obtaining images of multi-spectral nature was the calculation of indexes, such as the Normalized Difference Vegetation Index (NDVI), which could be covered with ease by means of the four detectors that the camera had installed. Overall, although the precision and data obtained from this UAV were remarkable, they are outside of the scope where our solution takes place.

In [21], Stuart Krause et al. put forward a UAV-based methodology to measure tree height for intensive forest monitoring, which is a major parameter to learn regarding tree growth, volume and biomass. Permanent Ground Control Points (GCPs) were set and measured with a Total Station, as UAV navigation with GNSS was likely to be ineffective in the environment of dense, high vegetation. Once data was collected, it was stored for later image indirect georeferencing and image pre-processing. In this case, the UAV-based tool used an “OctoXL 6S12 Octocopter mounted with a fixed lens Sony A7r RGB camera”. The camera itself had a 35 mm fixed lens manually focused at a hyper focal distance and was permanently fixed so as to avoid variations in focal length. The results showed that this was yet another application domain where UAVs could be used to obtain reliable information, even though its purpose was different from the one put forward in our paper. 

### 2.2. Open Issues

The reviewed literature shows that there are multiple ways to use UAVs in different environments for activities that are mostly related to data collection, either by using sensors or for monitoring purposes (which involve the usage of sophisticated cameras embedded as part of the UAV itself). However, as far as the application domain in our paper is concerned, there are very few examples on how UAVs can be used, and none of them are conceived as a fully functional prototype the way it has been built and described in this paper. Table 1 shows the advantages and drawbacks of each of the studied proposals in relation to the ideas that we put forward as authors of this study. 

In a more specific way, the open issues can be described as follows:

1. It is uncommon to use UAVs for the purposes and aims shown in this paper; many of the examples included deal with other aspects that, even when they show remarkable developments, do not intend to cover the purpose of measuring mobile network signals and parameters with a UAV. This creates a situation where there are multiple valid solutions that, unfortunately, cannot be applied or ported to the application domain shown in this paper.
2. Additionally, some of the papers studied in the reviewed literature rely on simulations to validate the hypotheses put forward. Although, depending on the context, this may be a valid methodology (it might even be that it is the only reasonable methodology to be used), it is usually more accurate and closer to reality using an actual prototype that will count on design, implementation and testing works to realize the contributions that are made to the existing state-of-the-art.
3. There is a degree of solution customization that is missing. This is due to the fact that, in several cases, instead of offering a system tailored for the purposes of the paper that is describing the research carried out, an already built UAV is used. This might come in useful in some cases, but it limits the flexibility and usability of the UAV when a testing prototype is deployed in the real world.
4. Finally, most of the solutions do not provide an end-user-friendly way of visualizing the information obtained by the UAVs used in the research works performed. Something, such as a GUI, is missing in many documents, which, from our point of view, would be an interesting option in order to offer retrieved data or conclusions in a more accessible manner.

Considering the range of applications and the literature previously studied, the hypothesis that should be put forward at this point, which is also within the application domain of the work presented in this paper, should be as follows: *is it possible to develop a solution capable of providing an inexpensive open-source tool to perform reliable mobile network measurements making use of Galileo as the GNSS?*

## 3. System Description

If the current state-of-the-art is taken into consideration, the improvements offered by the system put forward in the paper become clearer, especially when considering the contributions that have been put forward in Section 1:

1. Our built solution deals with an application domain that, judging from the reviewed literature, has not been given any relevant research so far. Therefore, we are providing research with a built prototype in an area of knowledge that has been almost previously untested, except for research studies that fall within this area but are focused on other objectives.
2. Use of an actual UAV enabled with Galileo as the Global Navigation Satellite System (GNSS). Rather than using an already existing UAV solution that has been built with purposes different from the ones put forward in this paper, a custom-made UAV has been developed for accurate sensing of mobile network signals. This customization has been used for two different purposes. On the one hand, a controller board (Navio2 [22]) has been used as a GNSS receptor for UAV positioning. Compared to other systems (GPS, GLONASS, Beidou, etc.), and as it will be described, Galileo offers a higher degree of accuracy and a more robust signal that offers a more exact positioning for any device making use of it. This comes in as extremely useful for this application domain, as high accuracy is required to perform mobile network measurements that are likely to change in terms of output in a relatively small location.
3. End-user-friendly capabilities have been provided as well. One of the three subsystems of the prototype is devoted to the development of a Graphical User Interface that will be able to display, in an accurate manner, what kind of data are being collected, where they were taken from and the meaning of them. This tool makes it easier to infer knowledge from the data acquired in any location the UAV flies.

These improvements have been summarized in Table 2.

As previously mentioned in the introduction, and to properly answer the hypothesis that has been put forward, a system was designed with the idea of developing a tool that, from a hardware and software perspective, is capable of measuring useful parameters of a network used for wireless communications in a mobile environment. Our proposal can be described as a Cyber-Physical System (CPS), combining three different subsystems interacting with each other. For each of the subsystems, a collection of functional and non-functional requirements were defined with the purpose of guaranteeing a certain level of quality and synergy among all of the parts that comprise the system. These parts can be defined in the following manner:

1. Open-source Unmanned Aerial Vehicle: this is the UAV that the authors of this paper have used to install the device used for measurements and to move it in a three-dimensional space.
2. Mobile Data Acquisition System (MDAS): this is used to collect data regarding the signal power levels in the areas where it is transported. It composed by a mobile phone and two applications: one to collect data and another to format it. It will be integrated as part of the hardware used as the UAV base station.
3. Graphical User Interface (GUI): this is a software program required to visualize the information that is shown to the end user.
4. Galileo GNSS: this is used as a pivotal part for this proposal, as it offers location features that are more accurate than the most widely used equivalent GNSS systems. This subsystem is taken for granted, as Galileo is already built and precedes the inception of the proposed system described in this paper. It was shown in [23] how the received Galileo signal (the one that will be used for the measurements) can be described in its Intermediate Frequency (IF) as:
  (1) $$Y_{k_{GAL-IF}}=(w_{k}e_{1B_{k}}sub_{B}-e_{1C_{k}}sub_{C})\;cos\;(2\pi f_{IF}t_{k}+\theta_{k})+N_{k}$$
  (2) $$sub_{B}=\alpha SC_{E1B_{ak}}+\beta SC_{E1B_{bk}}$$
  (3) $$sub_{C}=\alpha SC_{E1C_{ak}}+\beta SC_{E1C_{bk}}$$

**Figure 1:**
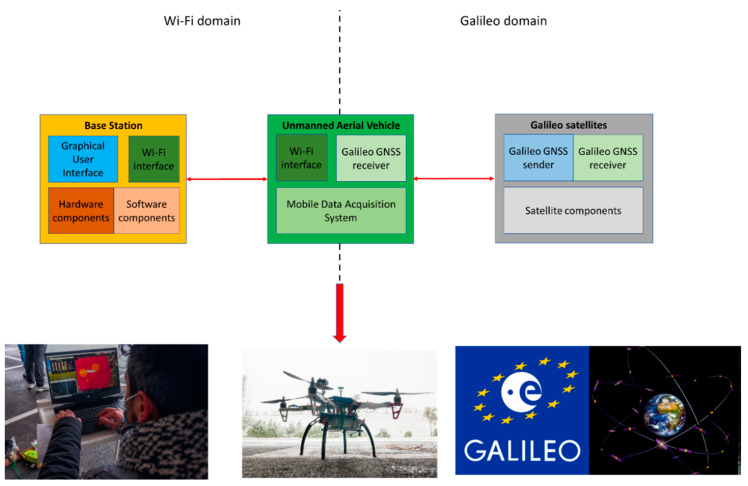
Components used in the proposed system.

In this set of equations, $w_{k}$ is an estimation of the Non-Return-to-Zero (NRZ) unpredictable symbol. $e_{1B_{k}}\;$ is the NRZ pseudo-random (PRN) sequence for the Galileo E1B Signal, whereas $e_{1C_{k}}$ is the NRZ pseudo-random (PRN) sequence for the Galileo E1C one. At the same time, $\alpha =\sqrt{\frac{10}{11}}$ and $\beta =\sqrt{\frac{1}{11}}$ and the $SC_{E1A}\left|B,a\right|b_{k}$. Finally, $N_{k}$ is the Additive White Gaussian Noise (AWGN) at the input of the receiver. Figure 1 shows how the subsystems interact with each other and what kind of interfaces are used to transfer information from one subsystem to another.

It must be noted that, despite the differentiations outlined in the figure, the subsystems have been integrated in a seamless manner. Furthermore, it can be seen how the MDAS is integrated as part of the UAV, as it will be implemented as a piece of hardware capable of sensing the data regarding mobile network signalling.

**Figure 2:**
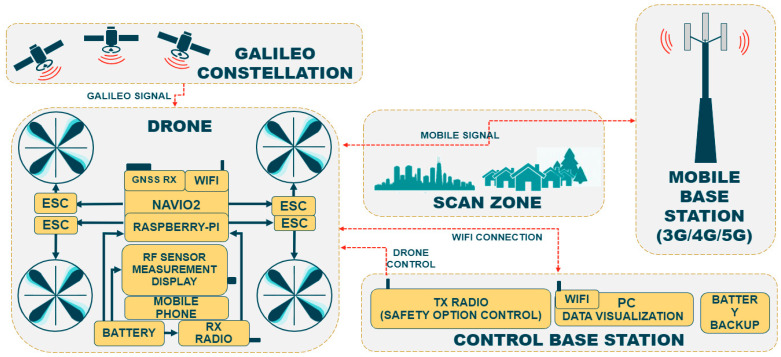
Relations and communication among major components of the system.

The three subsystems that have been previously mentioned as the ones used to build the proposed model (UAV, MDAS, GUI) interact with each other, as shown in the diagram displayed in Figure 2. The drone has been represented at the leftmost part of the diagram, with its most prominent parts displayed: wireless receptor (WIFI), Galileo receptor used by the autopilot for precise positioning (GNSS RX) and the MDAS equipment (radio frequency sensor measuring program and the mobile phone that is used for its installation and operation). It must be noted that the drone can connect to both the base station (composed by the radio transmitter for guided flight and the computer running the GUI used for data visualization) with its wireless module and to the Galileo GNSS via Navio2, which is the autopilot device used for flight control.

As can be seen, the presented subsystems make use of hardware and software developments. As in any other CPS, software elements have been built upon the hardware and their performance will trigger changes in hardware status. In this case, this will result in either the drone changing its flying direction, data being collected from the terminal or information being represented on the screen of the computer running the GUI. As mentioned before, Figure 2 shows how the drone makes use of the Galileo constellation as the GNSS service provider for UAV orientation and positioning, while, at the same time, being used for measurement accuracy. Note that a quadcopter has been chosen to represent the UAV structure.

Lastly, security features must also be considered for this development, as there are several threats that could be potentially exploited for cyberattacks that might result in an unwanted behaviour of the system, ranging from tampering with the collected data, from losing the UAV to some spurious third party. The attacks that could take place in the proposed development are as follows:

1. Base station monitoring: credentials used to access the base station could be leaked, or there could be other privacy failures that enable a spurious third party to monitor what kind of flight the UAV is carrying out.
2. UAV command spoofing: this attack is related to the previous one in the sense that it will require accessing the base station. Once the spurious party has managed to do so, it can alter the commands sent to the UAV to follow a different pattern or perform actions that could potentially lead to damaging the UAV to a greater or lesser extent.
3. Data tampering: in this case, information collected from the UAV flight can be tampered with in two locations: (a) either in the base station or (b) in the MDAS. It could lead to misinterpretations regarding network coverage or receiving/sending signals in mobile networks.
4. UAV hijacking: this cyberattack involves taking the UAV away from the location used for experimentation to somewhere where a spurious third party can take advantage of it. It will usually involve tampering with the Wi-Fi communications sent and received from the base station or with the GNSS signal; the latter is far less likely due to the extra security that it will make use of.

Consequently, security countermeasures must be undertaken to secure the data interchange and collection that takes place within the components of the developed system. Rather than writing any code that would involve the usage of cryptography, security is provided due to the use of several components in the proposed system, which already have built-in security features that will provide securitization to the whole system. Those elements are:

1. ArduPilot as the base station software: as described in the following section, ArduPilot has been used as the software for managing flights and UAV missions. Specifically, it makes use of a separate Mission Planner module used to conceive flights for specific missions that involve specific movements. As it was said in [24]: “*ArduPilot and Mission Planner have the ability to add security to over-the-air MAVLink transmissions by adding packet signing using an encrypted key*”, so this program can enable additional security features. The fact that ArduPilot is an open-source software development also aids in auditing command and information transfers ([25]) in case it is required. Thus, using ArduPilot and its Mission Planner will play a significant role in discouraging tampering with the UAV missions or the data collected.
2. Galileo as the GNSS: not only does it offer a higher degree of signal accuracy for device positioning (in our case, the UAV), but it also has additional security enabled by means of the Galileo Open Service Navigation Message Authentication (NMA), which provides “*an authentication mechanism that allows a GNSS receiver to verify the authenticity of the GNSS information and of the entity transmitting it, to ensure that it comes from a trusted source*” [26]. In this way, security in the communications between the GNSS and the UAV is upgraded and alterations in UAV missions or data collection become more difficult.
3. Wi-Fi as the wireless protocol used to establish communications between the UAV and the base station: the Wi-Fi protocol utilized for wireless communications is the 802.11ac iteration, which has security enabled in the signal sent throughout the used 5GHz frequency band. In this way, communications can be secured in the system at the physical level and will make it more difficult to tamper with the UAV’s behaviour or the received data.
4. Credentials for base station access: security capabilities can be added by providing an authentication mechanism that will filter the access to the hardware used, such as the base station. In this way, a first layer of security can be provided that will make it harder for spurious parties to access it, so that base station monitoring and data tampering can be prevented.

The security threats that might be faced by the developed system and the countermeasures taken to solve them have been summarized in Table 3.

Overall, the three subsystems that make up the tool displayed in this paper are described in the following sections.

### 3.1. Unmanned Aerial Vehicle

As previously explained, the system heavily relies on UAVs as a tool to ensure that measurements can be obtained from locations that could not be reached without it. In this way, the drone’s flying capabilities make it possible to cover extensive areas while requiring considerably lower amounts of time. What is more, as a single piece of equipment is used to take measurements rather than a team that could potentially require several people, it becomes a comparably inexpensive solution.

In order to design such a subsystem, it was necessary to conceive a series of functional and non-functional requirements that would cover all the aspects that would be required by the drone to be fully functional. This list is as shown in Table 4.

The UAV that was built makes use of several parts with different functionalities, as they are all required to be integrated in a single development. Some of these components are purely hardware oriented and consist of the parts used to build a physical UAV from scratch, so that it is tailored for the needs of the system. The other parts are the software associated with the UAV so that it can be used for actual mobile network monitoring missions.

#### 3.1.1. Hardware Components for the UAV

Since the main objective of the UAV is using it for mobile network measurement flights, hardware components were adapted to create a functional drone with the sensing capabilities to perform that duty. The most prominent components used to build the UAV were as follows:

1. Raspberry Pi 3B+: this is the component used to run the operating system used by the open-source drone to govern the other components [27]. This provides an entry point to modify every setting possible for drone flights, which is maximized by using the ArduPilot program, as explained in the software section of this paper. As far as this proposal is concerned, its pinout is used combined with the Navio2 Autopilot for flight guidance and coordination. The Raspberry 3B+ makes use of a processor with a clock frequency of 1.4 GHz and 1 GB of SDRAM memory, which offers enough computational capabilities for the purpose described in this paper, and, since it can provide IEEE 802.11.b/g/n/ac and Bluetooth connectivity, it has the required wireless interfaces for connectivity and data transfer.
2. Navio2: this is a controller board used, as in every UAV, to manage the drone flight on a real-time basis, making the UAV more stable and keeping it afloat in a safer way. Depending on the requirements, it makes use of either Linux-based Application Performance Monitoring (APM) or a tailored middleware to work with the Robot Operating System (ROS). This controller has a high resolution MS5611 barometer and 14 Pulse Width Modulation (PWM) output ports for control. One of its most prominent features is that the GNSS module (uBlox M8N) can use Galileo as the GNSS of choice. Considering its high accuracy level, and the fact that there are fewer applications that make use of Galileo (as opposed to, for example, GNSS), Galileo was used for UAV positioning.
3. Other components required for UAV assembly (batteries, drone frame kit, motors and propellers) were also required. They are described as follows:
  - Frame F450 [28]: For the frame or “skeleton” of the UAV, a Frame F450 with landing gear was chosen. This frame makes it possible to assemble all the parts on it and ensure stability to the drone. Among its main characteristics are resistance, lightness and a comparatively small size, which enable mounting several components while keeping low battery consumption benefits due to weight or stability.
  - MaxPRO 2650 Batteries [29]: 11.1V and 2650mAh batteries were used to power the UAV. They belong to the LiPo battery (Lithium polymer) family, which are the most used for drones since they allow fast discharges and can provide significant amounts of energy in a short time, in addition to being light and small compared to others.
  - Motors [30]: Set of four Emax 2213–935KV motors. These brushless UAV motors have 7.1 A as maximum current and are specially designed for 11.1V (3s) LiPo batteries, so they are a suitable choice for the drone battery. They include 10X4.5 propellers and have a thrust of 860g for each motor and 935KV (revolutions per minute/volt), which enable them to lift and manoeuvre the UAV without any issues.
  - FS-T6 programmable digital transmitter/receiver with six channels in 2.4 GHz [31]. Programming is easy and intuitive, which enables emergency or landing scenarios where a fast response is required. It has low power consumption and ultra-fast signal reaction with interference-free Automatic Frequency Hopping Digital System (AFHDS) technology. It works under a 500 Hz bandwidth, 1024 sensitivity, Liquid-Crystal Display (LCD), Pulse Position Modulation (PPM)/Pulse Code Modulation (PCM) security coding and supports up to 20 UAV models with this kind of receiver.

**Figure 3:**
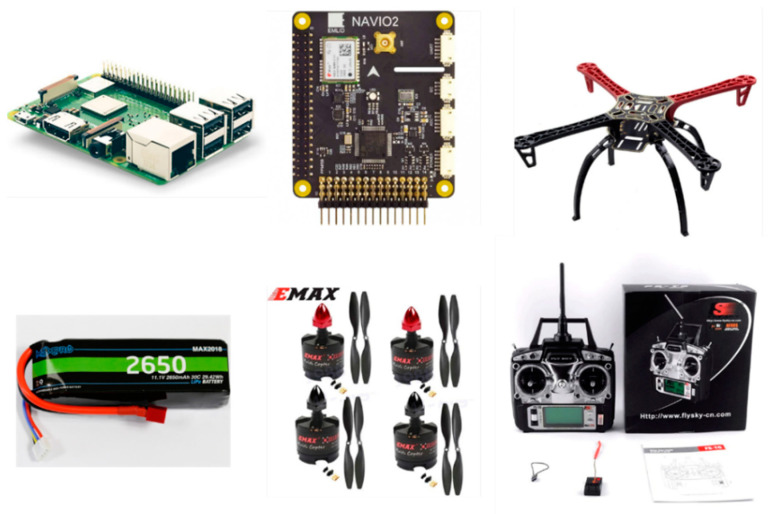
Hardware components used to build the drone.

These hardware components, while not adding any cutting edge or complex technology, are mandatory for the drone to be operational, otherwise the formulated hypothesis would have to be tested in a simulated environment, which the authors of the papers are trying to go beyond. The appearance of these components is displayed in Figure 3.

**Figure 4:**
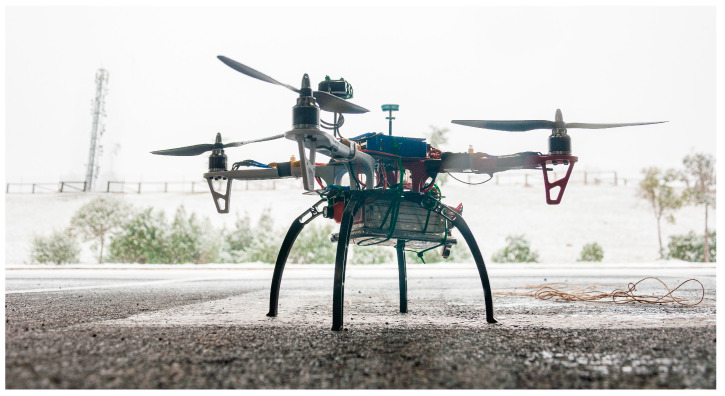
UAV built from scratch to meet the formulated requirements. Note the MDAS subsystem added to the bottom part.

All of these components were used to build up an enhanced version of the UAV as a way to have more suitable hardware for the tests carried out. The appearance of the prototype right before a test can be seen in Figure 4.

#### 3.1.2. Software Components for the UAV

**Figure 5:**
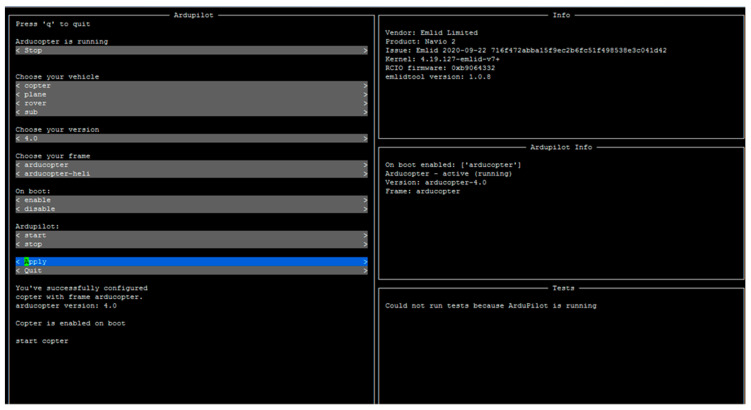
ArduPilot configuration details.

From a software perspective, there was another collection of tools that was used to integrate all of the components that the drone was made of. The most prominent were as follows:

1. Raspbian: this is the operating system run by the Raspberry Pi 3B+ mounted on the drone [32]. It is used for typical operating system duties: organizing memory accesses, providing a mechanism to manage the underlying hardware or, more importantly in the case of this proposal, providing a software ground from where to execute other programs that are more user oriented. Raspbian can make use of both a Graphical User Interface of its own or just a Command Line Interface, but its capability for running the AutoPilot planner is the most important functionality that it offers to the whole system presented in this paper.
2. ArduPilot: this is the flight planner used to manage all aspects of the taking off, in-air and landing navigation of the drone. It displays essential information on arming (turning on) or disarming the UAV and provides dashboards to recalibrate essential flight parameters of the drone, such as propeller regimes or commands to be carried out during flight. Although it has been conceived to be used in drones based on Arduino (hence the name ArduPilot), it is compatible with drones that make use of Raspbian and Raspberry Pi devices as the hardware backbone of the vehicle. ArduPilot works in the following manner: once it has been installed as a program, it will run as any other common piece of software on top of the operating system (in this case, Raspbian). Once it is executed, the vehicle operator will be given the option to configure the hardware, taking into account the kind of UAV (copter, rover, plane or even submarine) depending on what has been mounted and the controller set of the hardware (in our case, Navio2). Afterwards, as shown in Figure 5, further configuration details are completed.
3. Secure Shell (SSH) is used as the protocol and program used to communicate with the Raspberry Pi installed at the UAV from a terminal. For configuration and parameter changes, it is required to set the UAV to its desired parameters, so this protocol and its Command-Line-Interface-based tool are most useful.

The last step that was carried out for the UAV to fully serve the purposes conceived for Galileo was attaching the corresponding prototype of the designed MDAS system. Its main features and performance are described in the next subsection.

### 3.2. Mobile Data Acquisition System (MDAS)

The procedure to acquire information about mobile network coverage and overall signal power in a space requires not only using any kind of tool capable of manoeuvring throughout all of its locations within the said space, but also a way to collect information and the suitable hardware to be used. This is what is provided by the MDAS, which, as had happened in the previous subsystem, was built using a collection of hardware and software components. A thorough description of the requirements that were conceived for the MDAS is included in Table 5, which were deemed of major importance in order to have an accurate perspective of the functionalities that the MDAS should fulfil. It can be inferred how portability and signal information were the most critical aspects of the MDAS.

#### 3.2.1. Hardware Components for the MDAS

To collect information from the mobile network signal present in the area, there are two components that are needed: on the one hand, a hardware device capable of receiving such signals with its required features, and, on the other hand, a program able to provide a readable output of such signals in a suitable manner (for example, text files formatted according to the received information). From the point of view of our proposal, the hardware device to be used can consist of a smartphone, as they provide a portable piece of hardware where a plethora of programs oriented to our goals are executable. However, if a smartphone is to be used, it must have two key features related to this proposal:

1. It is of critical importance that the smartphone used is dual-band-enabled, so that the accuracy of the data obtained can be as great as possible.
2. The mobile phone should be compatible with Galileo as the GNSS. With this feature, the possible choices to use smartphones with those characteristics are significantly narrower. As already described, the use of Galileo as the GNSS enables a greater level of accuracy (Precise Point Positioning via High Accuracy Service, or PPP via HAS) and security (Navigation Message Authentication or NMA) that, to the best of our knowledge, cannot be matched with other global satellite systems, so there is an incentive to use it.

**Figure 6:**
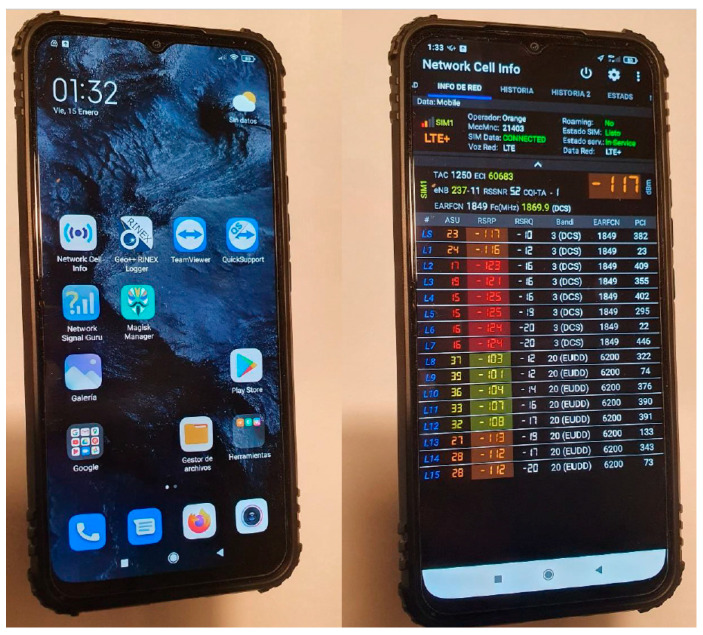
Xiaomi mi 10 lite (**left**) running the Network Cell Info program (**right**).

In the end, it was decided that the Xiaomi mi 10 lite [33] was the best possible option due to several reasons: (a) it is Galileo-friendly, (b) it has a dual-band receptor and (c) it is economically affordable. It could also make use of the software programs that had been installed to measure, as exact as possible, mobile network coverage values based on signal strength values. The smartphone is shown in Figure 6.

**Figure 7:**
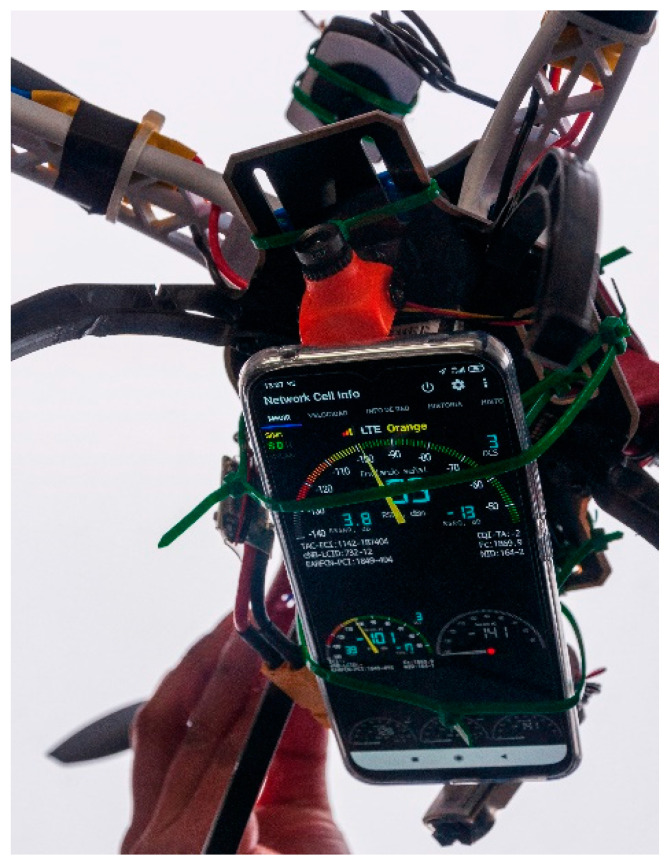
Xiaomi mounted with the UAV.

Therefore, the Xiaomi mi 10 lite smartphone was tied to the UAV that had been built as the device measuring the signal power and features obtained from the environment. The overall appearance of the phone can be seen in Figure 7.

#### 3.2.2. Software Components for the MDAS

As for the software, the main interest for the team was making use of a tool that made data acquisition possible regarding signal strength for the location where the smartphone was placed, regardless of it being on land or in mid-air. Another aspect to consider was the chipset of the hardware component used in the MDAS. Two apps were tested which required root privileges and with chipsets from the Qualcomm family. Fortunately, the Xiaomi mi 10 lite met these requirements by having a Qualcomm Snapdragon 765G Model Processor, so it was capable of running the required software.

While building a software application capable of measuring signal power received at a smartphone was considered (there are several different Java libraries used in the Android operating system that make that a possibility), software programs making use of those capabilities already exist and are fully functional. Among the programs available, there were two choices determined to be the most suitable: Network Cell Info [34] and Geo++ Rinex [35]. The former offers more direct usage and Comma Separated Values-formatted files as information output that are easy to port and transfer, so it was decided that it would be the program used. Network Cell Info can be described as a mobile and Wi-Fi network monitoring app with diagnostic and measurement tools compatible with 5G, LTE+, LTE, CDMA, WCDMA and GSM technologies. Among its main features are: (a) real-time monitoring of signals; (b) Internet speed tests, both in Wi-Fi and mobile networks; (c) Dual SIM support (a major feature for the smartphone used in the proposal); (d) a map with measurement information and connection status; (e) a location service (MLS, it provides an indicator of mobile locations on the Mozilla map); (f) capability to export measurements in Keyhole Markup Language 2.2, Mobile Location Service Geo-submit v.2, Cell Layout File v3, OpenCell Identifier Comma-Separated Values and CMWF data format; (g) device and SIM information. It offered a 1-year higher level subscription version at a cost of €3.29/year, so it was chosen due to its low price.

The option of exporting the data to use it later for analysis and uploading it to another system, along with its low cost compared to other alternatives, made this the choice for the proposed model. The CMWF Pro v.1 database exported with the pro version offers parameters such as the Received Signal Strength Indicator (RSSI), the carrier signal frequency, the device location accuracy in meters or channel quality indicators.

### 3.3. Graphical User Interface

The GUI was conceived to represent all of the information received from the Network Cell Info application run in the Xiaomi mi 10 Lite. It was successfully tested to run on a general-purpose computer that could also be used as the base station for all of the other aspects related to UAV flight planning. As with the other two subsystems, when this was designed, it was expected to have several functional and non-functional requirements that would define how it would behave and the parts that it would be made of. These requirements have been included in Table 6.

**Figure 8:**
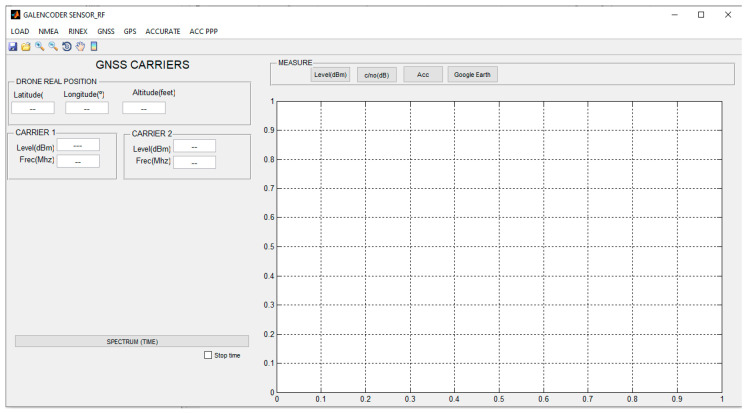
GUI appearance when idle.

The application was designed with MATLAB’s GUIDE tool [36], which can be described as the Integrated Development Environment of the Graphical User Interface. This tool was integrated into MATLAB, which can be described as a computer programming language that uses calculations and algorithms to analyse large amounts of data and present them in visually appealing formats. As far as our proposal is concerned, MATLAB and MATLAB´s GUIDE were used to develop the GUI, which was referred to as GALENCODER SENSOR RF. The appearance of the GUI developed for the project when it is idle is shown in Figure 8. It can be seen how it consists of two different areas: the left one used to characterize the information loaded, and the right one with the appearance of the different components of the signal output.

The developed GUI carries out the following functionalities:

1. Data load: the data are generated from the measurements collected by the radio frequency sensor (that is to say, the MDAS used to collect information) and can be stored either on an external device or on a server. Files use a Comma Separated Value (CSV) extension. Once located, the file containing the data will be uploaded to the GUI and used for further processing. Note that the operating system that is used belongs to the Windows suite and does not require any further sophistication: if a MATLAB image can be installed and run on a computer, it is capable of running the GUI. MATLAB images in other operating systems will result in using the GUI on them as well.
2. Data pre-processing: due to the noisy environment (in terms of radio frequency) where the measurements are taken, it is necessary to perform a prior analysis of the recorded data to detect anomalies in the recordings. Therefore, it is mandatory to identify outliers, missing values and/or perform a normalization to create a standard data structure that can be interpreted by the GUI. Processing and analysis of measurements involves numerical analysis of the measured values and interpretation of these results. From this process the main features of the measures are extracted, defined as: (a) power levels; (b) power-to-noise ratio levels; (c) frequencies of detected carriers; (d) measurement time intervals; (e) radiofrequency sensor position (latitude, longitude, elevation).
3. Signal measurement representation over a map: this is one of the most prominent functionalities of the GUI; it consists of displaying the numerical values extracted from the measurements in a friendly environment. This allows for a better understanding of the results with the aim of performing an analysis and interpretation in greater detail, and with the possibility of showing radiation levels on a real positioning map. There are several settings included with that option, such as downloading and showing maps, along with showing power levels over the map. This latter option makes it possible to distinguish coverage areas according to their power levels.

The subsystems that have been presented here prove that it is possible to perform a suitable implementation of the principles that have been put forward

## 4. System Testing and Performance

Specifications regarding performance are offered for the three different components that, from a technical point of view, have been developed for the whole system: (a) the Unmanned Aerial Vehicle (UAV) used to set a fight plan or fly the drone under given coordinates, (b) the Mobile Data Acquisition System (MDAS) used to collect information and (c) the Graphical User Interface (GUI), useful for data representation and user visualization. Each of these parts benefits from Galileo as the GNSS in their own way, either for data gathering or vehicle navigation. Overall, the proposed systems was built using the elements that have been previously presented. For them to work together and offer outputs that truly represent a combination of all of the features of the system, rather than isolated results, a series of experiments were carried out. These experiments were strongly related to the system features that have been described in the previous section and involved flying the drone and taking mobile network coverage measurements. Such measurements were taken by having the UAV execute flights with the MDAS subsystem already mounted on it, and being active in collecting information. As the UAV flew, data from the LTE communications were gathered by the application running on the smartphone and stored in a log file so they could be processed afterwards by the GUI used for data display. In any case, logs were also shown, so that the most relevant parameters could be visualized in this paper.

### 4.1. System Testing Considerations

The tests that were carried out followed several parameters to guarantee their usefulness.

1. Flight duration: the UAV flights were scheduled to last at least five minutes to obtain significant information about the drone’s flying capabilities, bearing in mind that some height and distance should be manageable as well. They were measured in ranges of hundreds of meters, so they guaranteed that the drone could fly significant distances to collect information.
2. Manoeuvring: the open-source drone built by the team members performed all possible movements in three dimensions (yaw, pitch, roll) to ensure that it had full mobility when in the air.
3. Data collection: the data that was collected was required to be significant for the purposes of the presented system. Therefore, it was required that it contained the parameters deemed as mandatory according to the non-functional requirements set in the previous section.

Furthermore, there were several external and internal elements associated with the tests that were performed. These were as follows:

1. Unfavourable weather conditions: the measurements to be taken relied on the built UAV being able to freely fly in a space as open as possible. Unfortunately, when the equipment associated with the MDAS was finally ready to be installed in the drone, there were severely unfavourable weather conditions. Consequently, UAV flights had to be planned and performed with extreme care not to damage the equipment that was being used. Despite these inconveniences, the authors of the paper were able to obtain accurate information about power levels from third and fourth generation mobile networks and map them with the coordinates that reflected where the UAV was when the measures were taken.
2. Information formatting data: as previously explained, the information collected is directly obtained from the Network Cell Info application for Android terminals. The data are obtained in Comma-Separated Values (CSV) format, and come with a plethora of parameters (the coordinates, altitude and power level of the strongest signal that is being measured) of different usability for the purpose of the proposal. The most useful data had to be picked from all the parameters to have it represented in a suitable manner.
3. Program coding: the GUI was programmed using MATLAB (Matrix Laboratory) as the programming language, due to its facilities to operate with matrix-formatted data. MATLAB imposed several syntax rules that must be followed, which, in the end, posed no issue at all.

### 4.2. System Deployment in the Real Scenario

**Figure 9:**
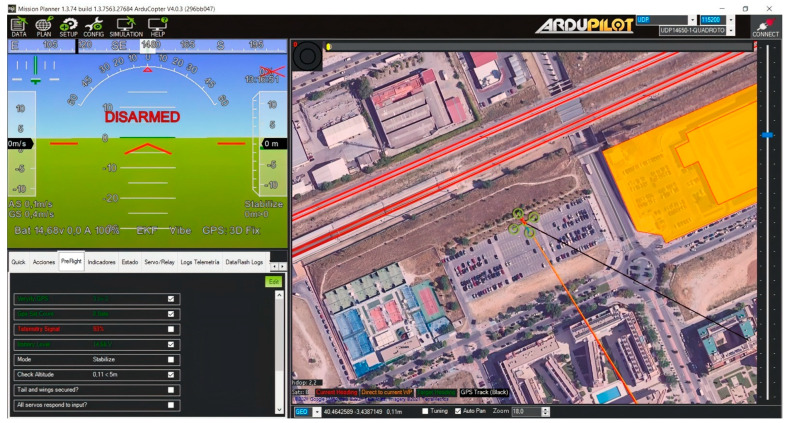
ArduPilot Mission Planner tool during one of the experiments.

The deployment of the whole system was carried out in an open space wide enough to perform experiments that would lead to valid, proven results that could be built to reach meaningful conclusions. An approach of incremental testing was followed, by which the team started performing lower key tests that, as the main objective, tested the behavior of the UAV itself, in case of software debugging of any kind, or if hardware refitting had to be done. The tests carried out have been listed as follows:

1. Open-air flight under normal conditions: the first experiment was aimed at testing what the UAV performance looked like under the regular conditions expected to be used. Expectations were that it would perform with next to no issues. The results showed no significant trouble during the UAV flight: both the hardware and software used (Figure 9 shows the information obtained from the AutoPilot program used for flight missions before the UAV took off) regularly performed and no reprogramming or hardware mounting were required.
2. Landing under normal conditions: in this case, the experiment dealt with landing the drone without any damage or issue resulting from a normal landing, so that it could be used without any restriction in its usefulness. Expectations were that this task could be carried out without any problems; the results confirmed these expectations.
3. Open-air flight during unfavourable weather conditions: during the experiments that were carried out, it became evident that, in order to be truly useful, the UAV would have to operate under weather conditions likely to be unfavorable. While it is not expected that the UAVs will have to work in a hostile environment that would damage the electronics (heavy rain, electrical storms), the UAV should be usable enough to guarantee that an occasional, light rainfall or wind drifts will not result in serious damage to the UAV that will incapacitate it to perform its duties. As described before, during the tests carried out with the UAV, it was proven that it could still work under such usage conditions, which had not been forecasted by the authors of this paper with enough importance during flight missions. Expectations were that the UAV would still be capable of normal flight under this kind of weather. While the results confirmed this, in terms of light rain and wind drifts, further testing was stopped in order to not damage the UAV.
4. Landing under unfavourable weather conditions: the UAV had to land in unfavourable weather conditions to prove that it could be recovered from flight missions without any problems. Expectations (the UAV could be recovered without having taken damage after the flight) were met again in this experiment.

Once it was proven that positive results had been obtained from the experiments, further flights were performed with the vehicle mounting the MDAS, so that not only the flight of the UAV and the MDAS combined could be tested, but also to collect information regarding power signals and their performance. Since the UAV used had already been successfully tested, there were several additional experiments carried out in order to test the whole infrastructure, along with the software developed for the proposal:

5. Information collection from mobile network signals: this is the main purpose of the system built and the reason why all the measurements are taken. In this case, the experiments were carried out to collect information related to 4G Long-Term Evolution (LTE) coverage in the area of testing and experimentation. The experiment consisted of, once the MDAS had been mounted to the UAV, setting the latter to fly and cover a large three-dimensional area with a speed and manoeuvrability that could not effectively be matched by a team of human operators. Expectations before performing this experiment contemplated the possibility of obtaining accurate information regarding mobile network signals.

**Figure 10:**
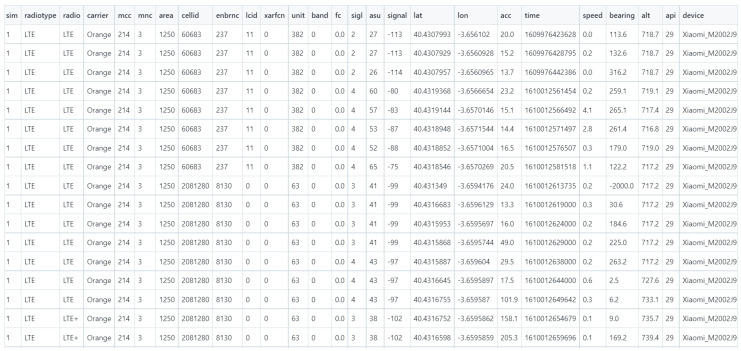
4G LTE data signal collected from the UAV mission.

The results showed, as depicted in Figure 10, that such data could be collected in an effective, fast and reliable manner, without having to incur the costs in terms of funds and time when using a team of people to collect that information from ground positions. When collected, data are formatted as Comma Separated Values (CSV) files, so data processing and visualization becomes simpler for common end users.

As can be seen from the previous image, there was a large collection of parameters that were obtained from the measuring UAV used to gather the data and the 4G that was sampled during the UAV flight. The ones that show important features related to our study are as follows:

- Sim: refers to the Subscriber Identity Module (SIM) card number used by the smartphone mounted as part of the MDAS subsystem running in combination with the UAV. There are two possible slots for SIM cards (1 and 2), depending on the local characteristics of the network mobile system for each country. For European countries, it is number 1.
- Radio type: references the kind of mobile network used when collecting information. In this case, it is LTE, as previously explained. Other options would be the Global System for Mobile (GSM) communication or Code-Division Multiple Access (CDMA).
- Radio: reference to the radio signal measured. Since there is no other possible option than LTE (GSM might use General Packet Radio Service (GPRS), for example) it has been labelled as LTE.
- Carrier: defines which network operator the used network belongs to. As can be seen in Figure 10, in this case it is Orange (France Telecom).
- MCC: an acronym that means Mobile Country Code (MCC), typical of mobile network systems. Since the experiments were run in Spain, it shows MCC 214.
- MNC: referred to as Mobile Network Code (MNC), which is the code used to identify the mobile network operator performing operations in a country. Since Orange/France Telecom is the network operator used for this experiment, and the data collection takes place in Spain (with a MCC of 214), it is identified as 3.
- Area: references the Tracking Area Code (TAC) used in LTE communications for area identification, which can range from 0 to 65,535. In the case of the measurements taken, the area is identified as 1250.
- cellid: a figure identifying the 4G cell used to collect information. The ones that appear in Figure 10 are 60,683 and 2,081,280, due to the fact that the UAV flew from one cell to another when collecting information.
- enbrnc: a node identifier that refers to evolved node base stations that are expected to behave according to the commands received from Radio Network Controllers (RNC) that are linked to the different cells used in the LTE system. Therefore, they have been identified as 237 and 8130 in Figure 10.
- lcid: an acronym that refers to the Logical Channel Identifier used in the corresponding Media Access Control Service Data Unit (MAC SDU) in communications in LTE. It changes several times (11 to 2, 1 and 0) in Figure 10.
- xarfcn: refers to the Absolute Radio-Frequency Channel Number used in the communications; 0 (as shown in Figure 10) is linked to band 1 in LTE.
- band: references the band used for mobile communications. While it is shown as zero in Figure 10, it actually means that the very first band in an array of available bands (starting at 0) is the one used here. Therefore, it refers to band 1, which is the first one available on LTE networks.
- sigl: refers to the signal level of power. The data collected and displayed in Figure 10 shows how levels go from 2 (the strongest one shown in the figure) to 4 (the weakest), so they summarize the signal power shown afterwards.
- ASU: an acronym for Arbitrary Strength Unit, and it is a parameter used for measurement mapping in LTE communications when signal power is taken into account. Thus, the highest ASU figures are the ones where signal power is stronger, and the weakest ones show lower ASU figures in comparison.
- signal: this value has the greatest importance for the purpose of the research in the paper, as it shows the strength of the measured power signal in decibels. In Figure 10, they are shown to cover a range between −75 dB (stronger, better signal quality) and −114 dB (weaker, worse signal quality).
- lat: refers to the latitude of the device used to measure the information from the 4G LTE signal, with the precision expected to be obtained from Galileo.
- lon: in a similar way, this feature is used to reflect the longitude of the device used for data measurements. Note that by using latitude and longitude combined, the location of the experiments performed and how the UAV moved around that area can be known.
- acc: this field refers to the expected accuracy of the measured positions or, from a different point of view, the likely positioning error to be measured. The values displayed in Figure 10 are shown in centimetres.
- time: a timestamp used to show the moment when the measurement was taken. The format used is Epoch, so it can be utilized to trace the moment when flying tests were carried out.
- speed: refers to the speed of the device used to calculate the measurements. The values for each piece of data collected appear in Figure 10; it must be considered that the information obtained is measured as scalar data rather than vectorial data, so only one field is provided. Measurements are taken in meters per second.
- bearing: refers to the azimuth angle (defined in this context as the angle between the object and the magnetic north of the Earth) taken during the MDAS measurements (which is directly linked to those taken by the UAV on which the MDAS is installed).
- alt: refers to the altitude that the MDAS was at when the measurements were taken. Figure 10 shows altitude values all well above 700 m; this is because the altitude measured takes 0 as sea level, and the testing location was placed in a land area already above more than 600 m over sea level.
- device: provides a description of the starting characters of the mobile phone that is used to collect information. Considering the smartphone that has been used as part of the MDAS (Xiaomi mi 10 Lite), it should come as no surprise that it is identified as Xiaomi_M2002J9G, which is the code used for such smartphones in mobile communications.

In addition to the pieces of data collected as shown in Figure 10, there were several other files with equivalent information extended in a more profuse manner in the GitHub project created for data storage. They are available in [37] (see in Supplementary Materials).

6. Combined UAV and MDAS landing. As previously determined, an experiment regarding how landing could be carried out with the whole measuring equipment mounted was performed. While the addition of hardware to the UAV presented some challenges in terms of how the latter would move (which included landing), in the end, we had the expectation that the drone would be usable with the MDAS. The obtained results corroborated this.

It must be stressed that, as explained in the abstract and as outlined in [37], the flights that were carried out during the UAV tests proved that the drone moved around more than 40 cells from the Orange network operator LTE system, as there were more than 40 cell identifiers collected as flight data. In addition to that, it must be mentioned how it was possible to retrieve information not only from the mobile network signal, but also from the nature of the mission. For example, the speed, altitude or trajectory of the drone can be known from having a look at the information both contained in Figure 10 and the data available at [37]. The latter shows that drone flight was carried out around 40.43 latitude and −3.65 longitude degrees as coordinates at an altitude over sea level of around 700 m, while travelling throughout more than 40 nano LTE cells and signal power between −75 and −113 dB (as shown in Figure 10). In a more specific manner, and considering the information available both in Figure 10 and the GitHub repository of [37], the following knowledge about the mission could be inferred:

1. UAV trajectory: while there were several tests carried out with the UAV flying different paths, there are several concrete aspects that should be mentioned about the flight missions that were carried out: the first flight took place from coordinates 40.4307993 degrees latitude and −3.656102 degrees longitude, hereinafter represented as (40.4307993, −3.656102)-, to (40.4640321, −3.4387723). Another was carried out from (40.4307993, −3.656102) to (40.4629463, −3.4404305) and a third took place from (40.4298989, −3.6573602) to (40.4522076, −3.4264832). Two more flights (with their information available in [37]) were carried out with similar positioning, which, in the end, provided information comparable to the previous three. Rather than using fully autonomous flight near a populated area, the latter flights were carried out by having a qualified drone pilot executing all of the manoeuvres, so the UAV trajectory was influenced by the pilot-controlled movements and there were no uniform shapes during the flight that were executed. To avoid any kind of legal issue, permission was requested to perform those flights.
2. UAV distance, height and speed while flying: there are some other pieces of information that can be inferred with regards to the UAV flight performance. Considering the coordinates previously provided, during the first flight, the UAV moved from two points separated by 18.78 kilometres, whereas, in the second and third flights, the starting and finishing points were separated by 18.61 and 19.71 kilometres, respectively. Height is visible in the field marked as “alt” in Figure 10, and it fluctuates between 640.7 and 718.7 m. As mentioned before, the existing ground elevation must be considered. For example, for terrestrial coordinates (40.4307993, −3.656102), it can be claimed that, due to the topographical characteristics of the Earth at that specific point, the altitude is 674 m. Since the flight altitude was registered as 718.7 m, the UAV was 718.7 − 674 = 44.7 m above ground. The finishing point for the first flight was 588 m. As for the second flight, the starting point was 665 m high and the last had an altitude of 558.19 m. The starting point of the third flight was also 665 m high and the last was located at an altitude of 569 m.

As far as the speed of the flight is concerned, it has already been shown how the parameter “speed” shows the speed of flight in metres per second every time a measurement was taken. Data collected from the MDAS showed that there were significant variations in speed during the flights taken; this is because speed is measured as a scalar magnitude (the speed module is given as a figure rather than the speed in every axis from a three-dimensional space) instead of a vectorial one. This also results in speed figures that might be significantly higher in absolute magnitudes than what would be obtained if speed was displayed in vectorial magnitudes, which results in drone speed being the least accurate parameter collected, even with some outlier values that are not physically possible. Nevertheless, the main purpose of the system presented was to provide a significantly faster way to collect data, rather than providing exact information on UAV flight. Figure 10 shows speed values ranging from staying still (0 m/s) to 4.1 m/s. As in previous cases, full information and details can be seen in [37].

**Figure 11:**
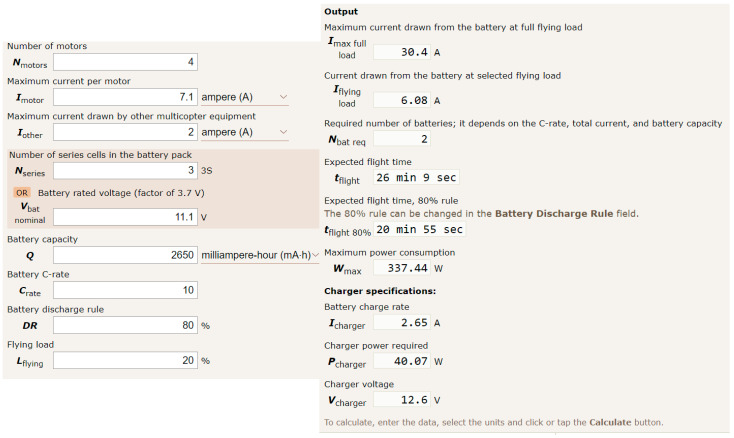
Information calculated for UAV flight, according to the UAV and battery capabilities.

3. Energy consumption of the UAV: as described before, a MaxPRO 2650 battery has been used to power the drone; it provides 11.1 Volts and a current of 2650 mAh. Considering the features related to the rotors (the most energy consuming element of the drone) and the other systems used in the UAV, a calculator was used to be aware of how long a flight could be and what energy and electricity consumption figures could be expected [38]. As shown in Figure 11, a maximum flight length of 26 min and 9 s can be obtained, with a more conservative figure of near 21 min for a safe flight that will deplete only 80% of the battery. Although these figures are largely theoretical, they were useful to get an idea of how long a flight could be. Additionally, information about energy consumption for the most important components of the drone could be calculated. Again, Figure 11 shows how 30.4 Amperes is the maximum current drawn from the battery at full flying load, whereas the Maximum power consumption expected from the UAV would be 337.44 Watts. Other compelling information (current drawn from the battery at selected flying load, charger specifications, etc.) has been obtained as well.

As the data were collected by using the Network Cell Info program, in the end, there was a significant degree of adjustability in the information retrieved, depending on the program that was used. Regardless of the application used in the MDAS, or the program written for such a purpose, there are several key information elements (signal power, location of the measurement, network operator, cell identifiers, etc.) that must be included in a mandatory manner.

**Figure 12:**
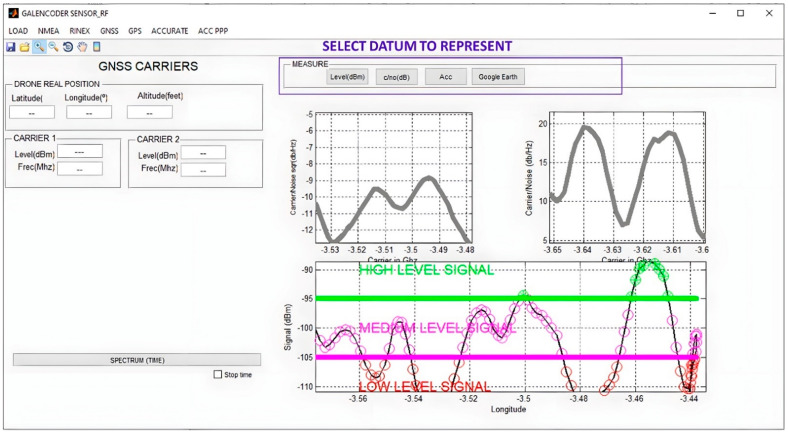
Information displayed by the GUI.

As can be inferred from the previous information, collecting information with UAV flights is plausible and the data collected is of interest. Furthermore, it can be obtained with a significantly cheaper and faster solution rather than using a team of technicians and/or engineers with their equipment. However, as appealing as this solution might be, it requires further processing after the information has been collected. The data collected must be visualized in a way that can be used by end users, regardless of their knowledge on the topics shown in this paper. As previously described, that is why a GUI was developed to display the most relevant data. The data collected under these experiments can be used as an example on how the GUI works, so that the information provided can be further extended: to begin with, the data collected from the CSV files obtained from the application installed in the smartphone used as MDAS will be loaded to the GUI by using a regular file explorer tab. Once the file has been loaded, information pre-processing will take place so that noise-related values are removed. Figure 12 shows how the GUI can display several pieces of relevant information, such as carrier-to-noise ratios for the two carriers used in data transmission for the Galileo GNSS and the signal level obtained as a representation of the collected data. Information shown in this figure is strictly related to the data collected by the drone, so it is a representation of the data shown in Figure 10, along with further data displayed in the GitHub link shown in [37]. As can be seen, data can be displayed in a user-friendly manner so that end users of the system will be able to infer knowledge from the drone missions in a fast way. There are also other actions that can be carried out with the GUI. As explained in Section 3, signal measurements can be represented over a map to better grasp the implications of the collected data in terms of actual coverage. As depicted in Figure 12, the information collected shows signal power measurements over a map, which reveals what locations or infrastructures are better covered by the 4G LTE signal and which ones have poorer signal values.

### 4.3. Result Discussion

The results obtained prove that the technological solution put forward in this paper is something to consider when collecting information from a mobile network. From the experiments performed, their expected results and what was obtained can be regarded as successful (Table 7 depicts these experiments). There were no significant deviations from the theoretically formulated results and what was obtained during testing activities.

**Figure 13:**
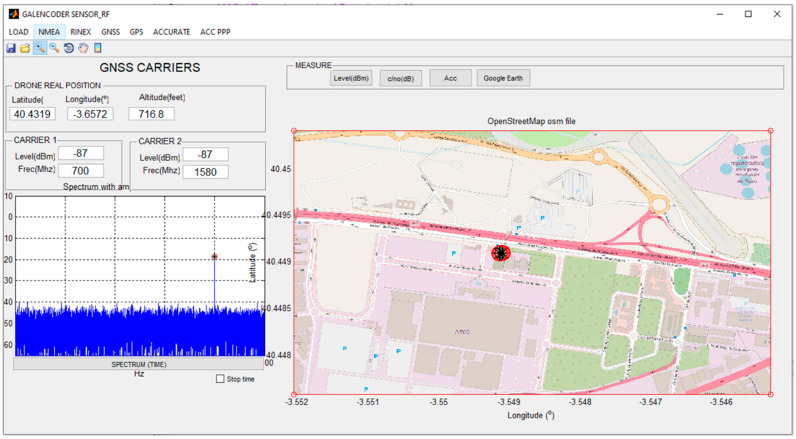
Map representation with mobile network signal values.

The information collected from the UAV flight (Figure 13) shows that, though there are significant values that can be regarded to be of major interest for end users and operators, they are still subject to minor errors in terms of location accuracy and signal power, especially if the values are too low. Pre-processing and filtering of information are also mandatory stages, as there might be collected data that are of little interest to end users or system operators.

Lastly, it must also be considered that the UAV used for this solution was tailored to the needs of the proposal, due to the fact that: (a) a Galileo-friendly UAV with the suitable chipset was required, and (b) a separated MDAS had to be built for data collection. Consequently, despite being valid as a prototype used to validate the formulated hypothesis, its usability as a commercial system could be limited when compared to already existing UAV solutions sold in the market. Further integration of the UAV and the MDAS is therefore desirable in future work.

## 5. Conclusions and Future Works

The authors of this paper built a drone with commercial off-the-shelf components capable of making use of several Galileo services that provide a comparative advantage when compared to other components that use different solutions that implement other components or GNSSs. Using a UAV as a starting point, additional parts were included to provide additional functionalities, like measuring signal levels in mobile network communications, data logging or information visualization via dashboards. The tests that were carried out confirmed a prototype that could make use of most of these characteristics.

At the end of Section 2, a hypothesis was formulated that was pivotal to the paper: is it possible to develop a solution capable of providing an inexpensive open-source tool to perform reliable mobile network measurements making use of Galileo as the GNSS? This hypothesis was taken as the main challenge by the authors of this paper due to the fact that, as had been previously explained, regular procedures to take mobile network measurements are slower (it takes a longer time to cover the area to be measured with a team of people, even if they are using vehicles, because they are confined to roads or have to be careful about rocky or hilly ground areas), more expensive (the cost of having an expert or a team of experts performing the measurements is usually higher than a UAV pilot and the UAV operating in an area) and less effective (even equipped with the suitable equipment, a team of humans cannot take as many precise measures in a three-dimensional space as a UAV flying to the specific points required). While the idea of using a UAV to take those measurements looked appealing on paper, it was necessary to implement a system that could be used for experimentation in the real world, as there were other parameters (weather, landing, flight, accuracy with Galileo GNSS, etc.) that could be scarcely determined with a theoretical approach. The work shown in this paper proved that the hypothesis formulated (that is to say, the challenge that was presented in this paper that the studied state-of-the-art failed to completely solve) could be answered in an affirmative manner and paves the way for further development in this application domain.

As far as future studies are concerned, it must be considered that the chosen solution proved its usability to acquire information from signal levels in the environment and could be upgraded in the future with the purpose of improving the accuracy of the data based on the positioning data that is obtained from Galileo. The use of the program Geo++ Rinex could be considered in future works as the logger application to obtain information, as it can be combined with the Galileo system regarding satellite positions that can be obtained from the NAVCAST server. In this way, updates regarding Galileo satellite positioning can be obtained. In addition to this, the possibility of building a UAV with an MDAS subsystem already integrated after its manufacturing could also be an important enhancement to the already existing solution.

## Figures and Tables

**Table 1:** Advantages and disadvantages shown in the studied UAV-based applications.

Authors	Advantages	Disadvantages
[10]	Usage of UAVs for wireless network measurements.	Simulated environment for some tailored use cases rather than actual prototype.
		
[11]	Antijam and accurate antenna parameters’ measurement framework is created.	No scope with open-source tool to perform mobile network measurements.
		
[12]	Autonomous Aerial Refuelling system designed.	Outside of the scope of this paper. Large UAV required for the application domain.
		
[13]	UAV can be used as a Mobile Measurement Platform (MMP).	No information or instructions regarding the application domain of this paper.
		
[14]	Proves that UAVs can be used for mobile network-related applications.	Focus oriented on the usability of UAVs as mobile base stations.
		
[15]	High precision aeromagnetic measurement equipment.	Requires large, oil-powered UAV.
		
[16]	UAV used for monitoring and parameter measurement in a natural environment.	Application domain far from the scope of this paper.
		
[17]	System created for Large-Scale Statistical Modelling of Air–to–Air Wireless UAV Channels.	Communications follow an Air-to-Air pattern; no GUI is provided.
		
[18]	Thorough study on how UAVs operate in congested wireless environments.	Application domain far from the scope of this paper.
		
[19]	Usage of UAV for measuring even rill and inter-rill erosion on the European loess belt.	Application domain far from the scope of this paper.
		
[20]	Usage of UAV for Normalized Difference Vegetation Index measurement	Application domain far from the scope of this paper.
		
[21]	UAV-based methodology to measure tree height for intensive forest monitoring	Application domain far from the scope of this paper.
		

**Table 2:** Solutions to the open issues provided by the proposed system.

Open Issue	Proposed Solution	Means Used for the Solution
No application domain research	Research using UAVs to collect information from mobile networks	Tailored UAV Galileo as GNSS
		
Lack of prototype deployment	Using an actual UAV whenever it is suitable for development and testing capabilities.	Tailored UAV Galileo GNSS
		
Lack of UAV tailoring for the application domain	Using an actual UAV adapted to the collection of mobile network data.	Tailored UAV Galileo GNSS End user capabilities (GUI)
		
Poor data visualization resources	Development of a GUI where significant information can be collected	End user capabilities (GUI)
		

**Table 3:** Security threats and their countermeasures.

Security Threat	Countermeasure
Base station monitoring	Credentials for base station access.
	
UAV command spoofing	Wi-Fi as the wireless protocol. Galileo as the GNSS. ArduPilot as the base station software.
Data tampering	Credentials for base station access. Wi-Fi as the wireless protocol. Galileo as the GNSS. ArduPilot as the base station software.
	
UAV hijacking	Wi-Fi as the wireless protocol. Galileo as the GNSS. ArduPilot as the base station software.
	

**Table 4:** Requirements for the UAV.

Requirement	Description
Functional Requirement 1	The UAV must be able to perform yaw, pitch and roll manoeuvres
Non-functional Requirement 1	The UAV must be able to have a mobile phone as the payload
Non-functional Requirement 2	The UAV must be able to stand off the ground for at least one minute
Non-functional Requirement 3	The UAV must use Galileo as the GNSS for positioning
Non-functional Requirement 4	The UAV must be able to fly away tens of meters

**Table 5:** MDAS requirements.

Requirement	Description
Functional Requirement 1	The MDAS must be portable so it can be installed in a UAV.
Non-functional Requirement 1	The MDAS must be fully operational with a regular smartphone.
Non-functional Requirement 2	The MDAS must be able to collect information about coordinates.
Non-functional Requirement 3	The MDAS must be able to collect information about altitude.
Non-functional Requirement 4	The MDAS must be able to obtain information about signal power levels.
Non-functional Requirement 5	The MDAS must be able to run the program used for signal logging.

**Table 6:** GUI requirements.

Requirement	Description
Functional Requirement 1	The GUI must have a dashboard where signal information can be visualized.
Functional Requirement 2	The GUI must be able to visualize data on a map.
Non-functional Requirement 1	The GUI must run on any regular laptop without having any performance issues.
Non-functional Requirement 2	The GUI must show accurate data about coordinates.
Non-functional Requirement 3	The GUI must show accurate data about altitude.
Non-functional Requirement 4	The GUI must show accurate data about mobile network signals in terms of power.

**Table 7:** Experiments carried out and comparison between expected and obtained results.

Experiment Performed	Expected Result	Obtained Result. Deviations
Open-air flight under normal conditions	Regular flight of the UAV	Regular flight of the UAV
		
Landing under normal conditions	Regular landing of the UAV	Regular landing of the UAV
		
Open-air flight during unfavourable weather conditions	Acceptable flight of the UAV	Acceptable flight of the UAV
		
Landing under unfavourable weather conditions	Acceptable landing of the UAV	Acceptable landing of the UAV
		
Data collection from UAV	Information collected from the mobile network	Files with information from the mobile network
		
Combined UAV and MDAS landing	Regular landing of the UAV	Regular landing of the UAV
		.

## Data Availability

As describe before, the files containing the MDAS logs, as well as the source code for the Graphical User Interface, can be found in https://github.com/jrodrimo/GALENCODER (accessed on 25 December 2022).

## Supplementary Materials

The files that have been created about the tests carried out and the displayed information are available in https://github.com/jrodrimo/GALENCODER/tree/main/logs. MATLAB files containing the source code of the GUI are included there as well (accessed on 27 December 2022).
